# Preparing for the Next Wave of COVID-19: Resilience in the Face of a Spreading Pandemic

**DOI:** 10.3390/ijerph17114098

**Published:** 2020-06-08

**Authors:** Gerald Steiner, Lukas Zenk, Eva Schernhammer

**Affiliations:** 1 Department of Knowledge and Communication Management, Faculty of Business and Globalization, Danube University Krems, 3500 Krems an der Donau, Austria; gerald.steiner@donau-uni.ac.at (G.S.); lukas.zenk@donau-uni.ac.at (L.Z.); 2 Complexity Science Hub Vienna, 1090 Vienna, Austria; 3 Department of Epidemiology, Center for Public Health, Medical University of Vienna, 1090 Vienna, Austria; 4 Channing Division of Network Medicine, Harvard Medical School, Boston, MA 02115, USA

**Keywords:** pandemic spread, healthcare workers, COVID-19, network scenarios

## Abstract

COVID-19 painfully demonstrates how little resilience our societies have to novel viruses. Societies, decision makers, and scientists lack (1) a comprehensive understanding of the complexity of viral outbreaks and their impact on society; (2) intervention portfolios; and (3) a global crisis and resilience policy, all of which are required to develop appropriate measures and to improve societal resilience. We highlight COVID-19 immunity as one key benchmark in preparation for the next wave of the pandemic. Specifically, using network scenarios, we demonstrate the substantial advantage of reintegrating health care workers with acquired COVID-19 immunity in epidemic hotspots, which would not only enable their safe contribution to the health care system but also drastically contain further spread.

## 1. Need for Integrated Knowledge

The multi-dimensional corollaries of COVID-19 comprise effects on health and wellbeing, as well as economic, financial, political, legal, institutional, sociocultural, technological, and ecological consequences within societal systems [1]. A sufficient understanding of such highly interlinked system influences and uncertainties requires broad interdisciplinary cooperation and a systems science approach as a common platform for knowledge integration and discourse between disciplines (i.e., interdisciplinarity), as well as the involvement of citizens and relevant experts from practice (i.e., transdisciplinarity).

COVID-19 painfully demonstrates that societies, decision makers, and scientists lack (1) a comprehensive understanding of the complexity of viral outbreaks and their impact on society; (2) effective intervention portfolios; and (3) a global crisis and resilience policy, in order to develop appropriate measures and thereby enhance societal resilience. As a crucial first step, we highlight the necessary understanding, and develop initial scenarios regarding COVID-19 immunity as one key benchmark for a possible intervention portfolio, building on the authors’ combined expertise and integrated knowledge in systems science, network science, and epidemiology.

## 2. Uncertainties in Viral Outbreaks

While immersed in the first wave of the COVID-19 pandemic, much uncertainty surrounding the behavior of this pathogen remains. A few guiding principles can help define and better understand the severity and uncertainties of any epidemic outbreak: the (1) basic reproduction number R_0_; (2) lethality associated with the pathogen (e.g., the case fatality rate, measured as the proportion of infected persons who die from the infection); (3) the duration of its incubation period (e.g., the time between exposure to the infectious agent and the appearance of first symptoms); and (4) the number of individuals immune to the pathogen in a given population.

The basic reproduction number R_0_ provides a sense of the speed at which a disease spreads, i.e., its contagiousness. If one person, on average, infects just one other person, R_0_ = 1. If one person infects on average two other persons, R_0_ = 2, and so forth. Any R_0_ > 1 has the potential to lead to an outbreak, as the number of cases is prone to increase. For COVID-19, R_0_ is currently assumed to fall between 1.5–3.5 [2,3]. COVID-19′s R_0_ contrasts with the R_0_ of, for example, measles (~12.0) [4], the flu (~1.2–2.2) [5], or Ebola (2.0) [6]. This brings us to the second guiding principle to appraise the severity of an outbreak, the lethality of a given pathogen. For example, while the high R_0_ of measles implies that many people will become infected, its mortality (e.g., case fatality) rate is low (~0.2%), in contrast to Ebola, which has a much smaller R_0_ and hence is less contagious, but which is far deadlier (mortality rate 22.0–88.0%). For COVID-19, projections from China [7], with some variation [8], indicate a 2.3% case fatality rate. Third, the incubation period of COVID-19 (median 5.1 days, spanning up to 15.6 days [9]) is unfavorable compared to that of 1–2 days for the flu. Fourth, because COVID-19 is new, the virus can potentially spread unchecked in a global population with essentially 100% susceptibility (e.g., due to no source of prior immunity).

Together, these factors implicate COVID-19 as a serious global threat. Mitigating factors that could slow the spread of transmission and lower the R_0_ include the availability of measures to control the disease, e.g., vaccines, or the level of immunity acquired in a population. Of note, the proportion of susceptible in the population has implications for the R_0_; the term “effective” reproduction number, eR_0_, factors population susceptibility into the estimation of the number of new individuals that an infected person can infect. eR_0_ can readily be estimated using the formula eR_0_ = R_0_x, where x is the fraction of the host population that is susceptible. For example, if R_0_ for measles is 12 where half of the population is immune, the eR_0_ for measles is 12 × 0.5 = 6 (a single case of measles would produce an average of 6, not 12, new secondary cases). Conversely, the herd immunity threshold (R_0_ − 1)/R_0_ is the proportion of a population that must be immune in order for an infectious disease to reach R_0_ < 1 and thus stopping its spread. For COVID-19 to reach R_0_ < 1, with a conservatively estimated R_0_ of 1.5, 33% of the population must be immune, and if R_0_ = 3.5, at least 71% of a given population must be immune for the infection to stop spreading. Additional influencing factors include, e.g., environmental factors, population density, cultural norms, health status, or average population age.

Experience from previous outbreaks suggests another important phenomenon for the derivation of R_0_: the super-spreaders [10,11]. Limited evidence is available to address how these super-spreaders could be identified to include them in, and thus refine, predictive models [10,12,13]. Several super-spreader factors have been suggested to define their infectivity, including host factors (e.g., their immune system or behavior); pathogen factors (e.g., virulence); and environmental factors (e.g., crowding) [10].

## 3. Network Scenarios: The Super-Spreaders

From a social network perspective, individuals are embedded in diverse social interactions [14], which evolve over time and can be simulated in network scenarios. In the context of viral spreads, the most crucial social interaction is the physical contact of people and its impact on the probability of viral transmission [15]. In this regard, health care providers, including hospitals or care facilities, are central. Health care professionals (HCPs), of whom up to 20% are said to be infected with COVID-19 [16], can be considered super-spreaders [17,18]; they have close physical contact with a high number of persons (“degree centrality”), particularly with other highly-connected HCPs (“eigenvector centrality”), as well as—importantly—with patients in poor health, in a setting where physical distancing is not feasible. Moreover, they are often required to continue working during outbreaks, which increases the risk of infection and downstream consequences.

**Figure 1:**
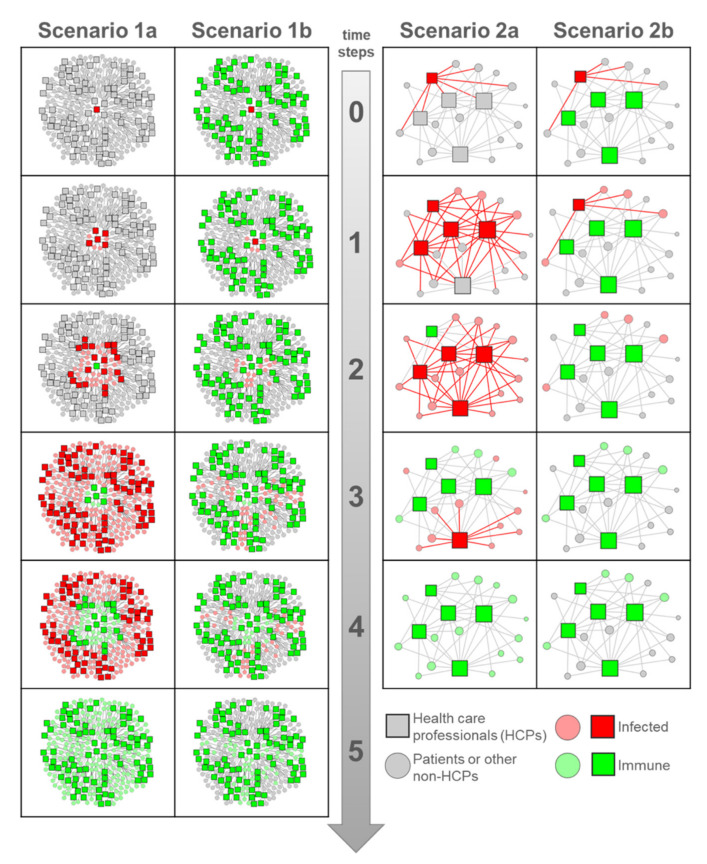
Network scenarios of the spread and immunization at the beginning of an infectious disease. Each time step indicates a time period in which one person infects R_0_ other persons. Health care professionals (HCPs) are represented as rectangles, and non-HCPs are represented as circles. The colors depict the health status: red (infected), green (immune), gray (neither infected nor immune). In condition (a), HCPs can be infected, and, in condition (b), HCPs cannot be infected. Scenario 1: The organization of a health care provider, including HCPs and non-HCPs. In Scenario 1a, all 265 persons were infected over time compared to only 53 persons in Scenario 1b. Scenario 2: Department within a health care provider including HCPs and patients. The size of the nodes indicates the number of contacts to other nodes. In Scenario 2a, all 20 persons were infected compared to only 4 patients in Scenario 2b. Created with the software Visone [19] (see Supplementary Materials, for the external xlsx data base that was used for graphics).

In the following, we show two scenarios of social networks in the context of health care providers at the beginning of the viral spread and over time with increasing immunization (see Figure 1). In the first scenario, we focus on an entire organization, in the second on a small department. For each scenario, we distinguish between two conditions. Under condition (a), HCPs can be infected by the virus (e.g., because they have not acquired immunity yet or were not otherwise well protected). Under condition (b), HCPs cannot be infected or infect others (e.g., due to previous infection, vaccination—which does not exist for COVID-19 so far—and/or taking appropriate protective measures, such as using personal protective equipment (PPE)).

In Scenario 1 (ego networks), we assume that various people are at a health care provider (e.g., a hospital), including HCPs (e.g., doctors, nurses, caregivers) and non-HCPs (e.g., patients, visitors, hospital staff etc.). As a starting point (time step = 0), one HCP was infected with COVID-19. In each of the following time steps (time steps 1–5; e.g., weeks), an HCP infects as a super-spreader 8 other persons (4 HCPs and 4 non-HCPs) and a non-HCP infects 3 other non-HCPs (hypothetical R_0_ = 3 for COVID-19). In Scenario 1a, a total of 265 persons were ultimately infected by the first HCP after only three time steps, including 85 HCPs (the number of deaths is not considered in this model). Without the here-assumed system boundary of a 3-step ego network, the beginning exponential growth of the virus would further increase. In Scenario 1b, the virus is blocked by the immune HCPs and cannot spread in a similar manner. In total, only 53 persons were infected, including the first infected HCP.

In Scenario 2 (core periphery networks), we further illustrate the power of immunity in HCPs, focusing on a small department within a given healthcare provider, e.g., an intensive care unit including HCPs (only doctors and nurses) and non-HCPs (only patients). In this scenario, HCPs are connected to other HCPs and patients; patients are separated from each other and are only connected to HCPs. Due to the physical proximity, we assume that the virus will spread through the direct contacts. In Scenario 2a, HCPs are not immune or immunized, and all 20 persons are infected after three time steps. In Scenario 2b, we assume that all HCPs with the exception of the first infected HCP, are immune. The infected HCP infects, initially, 4 patients, but no other HCPs. As illustrated, even in the next time steps, no more persons are additionally infected, and by the subsequent step, all persons have either acquired immunity or have not become infected.

## 4. From Super-Spreaders to Virus Task Force

As demonstrated in our simplified network scenarios, immunized (or otherwise well-protected) super-spreaders can be critical for effective local and possibly (in the case of pandemic spread) global health care.

**Figure 2:**
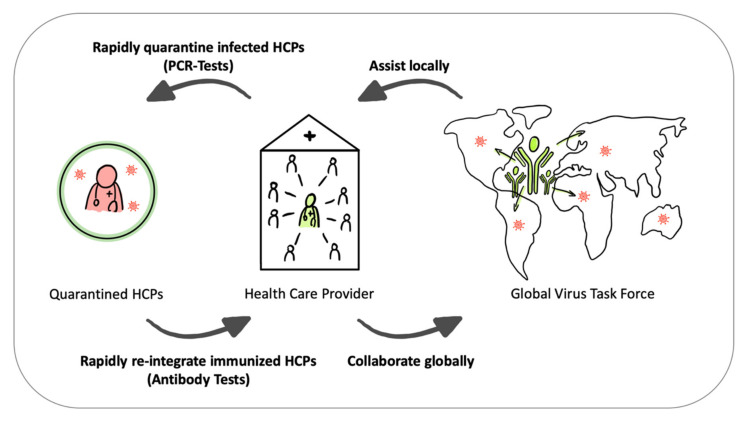
Virus task force without borders. On the left, more PCR tests allow for the rapid quarantining of infected HCPs, and antibody tests identify immunized HCPs to reintegrate them into the health care systems. On the right, international immune HCPs collaborate globally and assist locally in epidemic hotspots, as pathogens do not stop at national borders.

Reverse transcription polymerase chain reaction (RT-PCR) and antibody tests would enable the identification of infected HCPs and those who acquired immunity to either (1) rapidly quarantine them, thus preventing the infection (and quarantine) of a large number of additional HCPs as well as patients, or (2) help reintegrate them into epidemic hotspots where they can now safely contribute to the health care system. As one measure within a comprehensive intervention portfolio, immune HCPs could serve as a “virus task force without borders” to help minimize the infection of additional super-spreaders (e.g., susceptible HCPs) and thereby ward off the pandemic spread in other regions where they are needed (see Figure 2).

In our network scenarios, we oversimplify real-world problems and the complexity of social interactions (for example, we do not include weighted interactions, probabilities of transmissions, or event-based interactions over time, and solid empirical data for COVID-19 are lacking to enable a precise parameterization of the models). However, they represent a first attempt for inter- and transdisciplinary explorations of network analytical social interactions in vulnerable subsystems, such as health care providers. In the future, more in-depth systemic analyses with empirical data collected over time are needed. Ultimately, such analyses could inform sophisticated resilience management, which is urgently needed to improve society’s capacity to cope with similar threats in the future.

## 5. Conclusions

Clearly, in our interconnected world, it will become more difficult to stop pathogens at national borders; cooperation is more important than ever to tackle future societal challenges in an inter- and transdisciplinary manner. By integrating the various capacities and adaptability of local, regional, and international societal systems to absorb and recover from major adverse events, such resilience management extends far beyond current risk management [1,20].

## Supplementary Materials

The following are available online at https://www.mdpi.com/1660-4601/17/11/4098/s1. We include S1_Network_Graphs, i.e., the external xlsx data base for graphics in Figure 1.
