# Supplementary material for: Preparing for the Next Wave of COVID-19: Resilience in the Face of a Spreading Pandemic
Source: Int J Environ Res Public Health. 2020 Jun 8;17(11):4098. doi: 10.3390/ijerph17114098 (PMC7312045; doi:10.3390/ijerph17114098)
Supplement: Supplementary file 1 [file ijerph-17-04098-s001.pdf]

Calculations for the manuscript

Scenario1a

Total persons	265
Total HCPs	85

Scenario1b

Infected	53

Scenario2

Infected patients	4
